# National Insurance Committees: Injustice and Unreason

**Published:** 1915-09-11

**Authors:** 


### NATIONAL INSURANCE COMMITTEES.

### Injustice and Unreason.

We have received the following important letter from "Hospital Manager." We invite correspondence: "As The Hospital has so often pointed out, the National Insurance Act was an attempt to deal with sickness and disease throughout the United Kingdom which left entirely out of consideration the need of providing for insured persons requiring institutional treatment for general medical and surgical conditions. This was an evasion of responsibility which made the National Insurance Act of comparatively little usefulness as a remedial Act, for in reality it became a measure which in its general provisions dealt only with minor cases of sickness. The Hospital submitted at the time of the passing of the Act that working men and women had been and were prepared to provide from their own resources for such treatment, but they were not able to provide for sickness of an urgent or acute nature, requiring institutional treatment or skilled nursing. Had the Insurance Act helped them in this provision it would have been a great measure for the good of working men and women.

### Injustice to the Hospitals.

"The National Insurance Commissioners, the body charged with administering the Act, endeavoured to condone the limitations they placed upon the Act by assuming that the voluntary hospitals could be relied on to afford hospital treatment to insured persons on the same terms and conditions on which it was open to members of the general community to obtain such treatment. This assumption offers an interesting reflection. The Commissioners rely on the one hand on the good will of the voluntary hospitals to enable the National Insurance Act to become effective in adequately providing for illness and disease, and they are quite willing for insured persons to seek their treatment from the voluntary hospitals, without any question of suitability of staff, equipment, etc. But in the case of tuberculosis treatment provided for insured members under another section of the Act, the Commissioners require the same voluntary hospitals to be "approved" as to suitability of staff, equipment, etc., before Insurance Committees and Council authorities can enter into agreements with them for the treatment of tuberculosis, either generally or in any particular form.

### The Position of the Tuberculous Patient.

"It follows that this attitude produces several striking anomalies; of hospitals, on the one hand, being encouraged to undertake the general medical and surgical treatment of insured persons, but discouraged, on the other hand, from undertaking the surgical treatment of tuberculosis, although, be it noted, adequate alternative arrangements are not made for the provision of such treatment; of convalescent homes with resident matrons, but without resident doctors, being "approved" for the reception and treatment of cases of tuberculosis, while the voluntary hospitals, which are the nation's training ground for the supply of qualified surgeons and trained nurses, and which have been the only means of providing for the surgical treatment of tuberculous disease amongst the poor for generations past, are required to be "approved" for treatment of tuberculosis cases, although, as we have said, no other provision is made.

### Facing-Both-Ways Commissioners.

"The National Insurance Commissioners are ready, in other words, for the hospitals to undertake without approval the gratuitous treatment of insured persons for general medical and surgical conditions, which the Act should provide, but does not, but they are unwilling for the same institutions to undertake the treatment of tuberculosis as part of the arrangements for the administration of the Act without approval as to staff, equipment, etc., although they have no unwillingness to allow tuberculous patients to be transferred from "approved" convalescent homes for surgical treatment in the hospitals. This Gilbertian situation calls for some comment as to what the "approval" of institutions means. The periodical inspection of institutions to judge of their suitability for the treatment of tuberculosis seems a simple and innocent arrangement enough, but I question if the experience of hospital governors warrants them in the assumption that this periodical inspection may always be of the formal nature implied—not unlikely in a few years hospital governors would find their institutions conditionally approved, subject, for example, to the provision of a certain number of beds for tuberculosis treatment, to the addition of extensive balconies, or to other conditions being carried out.

### Government Exploitation Endeavour.

"But even if hospital governors did seek for their institutions to become "approved" they would be met with this difficulty, that the approval could only be of the hospital buildings, and could not include the approval of honorary medical services. To be quite frank, I question if any institution is justified in entering into a financial arrangement with a Government authority administering medical benefits in return for monetary contributions, for the receipt of money arising out of the treatment voluntarily rendered by honorary medical and surgical officers. Such a course would alter the whole basis of voluntary hospital work. Voluntary hospitals in the past have been too much exploited by Government and other Departments. The treatment of school children, for example, has been a source of endless contention between Education Committees and hospitals, and will continue to be until Education Committees not only provide out of the educational grants they receive for medical inspection, but efficient medical treatment, for which treatment they are still largely dependent upon the voluntary hospitals.

Defectiveness of Approved Institutions.

"Town and County Councils are charged with the provision of treatment for tuberculosis under the National Insurance Act: they approve convalescent homes and other buildings for the purpose, without making the slightest attempt to engage a staff to enable them to provide adequate treatment for all tuberculous conditions, with the inevitable result that in effect the voluntary hospitals are called upon to provide for the deficiencies of approved institutions. I know of cases which have been admitted to approved institutions for tuberculosis treatment, where the treatment has been no more than the provision of skilled nursing and change of air, adequate treatment has only been secured when the patients have been removed to voluntary institutions where operative treatment is available. Insurance Committees, in fact, seldom in my opinion look beyond the financial side of the work they undertake. Thus the great importance of adequate institutional treatment for surgical tuberculosis has so far received scanty recognition.

"The failure to provide adequate medical benefit and sanatorium benefit to insured persons calls, in my opinion, for condemnation, and I hope when this terrible war is over and peace is restored that attention will be focussed upon the urgent need for a complete revision of the arrangements at present made for medical and surgical treatment by Insurance Committees in carrying out the provisions of the National Insurance Act."

